# An interorganizational task force for disasters affecting US blood supply

**DOI:** 10.1111/vox.70166

**Published:** 2025-12-09

**Authors:** Glenn Ramsey, Frank Berry

**Affiliations:** ^1^ Department of Pathology, Feinberg School of Medicine Northwestern University Chicago Illinois USA; ^2^ Association for the Advancement of Blood & Biotherapies Bethesda Maryland USA

**Keywords:** blood banks, disaster planning, disasters, mass casualty events

## Abstract

**Background and Objectives:**

The US Interorganizational Task Force for Domestic Disasters and Acts of Terrorism (Interorganizational Disaster Task Force [IDTF]) is coordinated by the Association for the Advancement of Blood & Biotherapies (AABB). The IDTF was founded in 2001 in response to the 9/11 attack and is part of the US Department of Homeland Security's National Response Framework for disaster responses. We sought to set out the objectives, methods and activities of the IDTF.

**Materials and Methods:**

We reviewed the organization and history of the IDTF and representative disaster responses coordinated by the IDTF.

**Results:**

The IDTF is composed of representatives from five civilian and military blood organizations, five key healthcare organizations and liaisons from the US Department of Health and Human Services (HHS) and two HHS agencies. The IDTF promotes disaster preparation by blood centres and transfusion services and communicates weekly with its federal partners on the disaster readiness of the US blood supply. During disasters, which often interrupt regional donor operations, the IDTF helps in guiding national blood collection efforts, provides data‐based messages to HHS and the public on blood donation needs and coordinates inter‐regional blood transport and blood‐supply infrastructure support. The IDTF has been activated for large‐scale weather events, Zika and COVID‐19 epidemics, cyberattacks and an earthquake (Haiti).

**Conclusion:**

Because the US blood supply is not under a centralized authority, the IDTF is a unique and critical forum for collaborative private–public sector partnership to overcome disaster‐related challenges to blood availability.

**Highlights**

- The US Interorganizational Disaster Task Force (IDTF) promotes blood banking disaster preparation, monitors the disaster readiness of the US blood supply and coordinates blood donor messaging and inter‐regional blood supply responses in emergencies.
- IDTF members are key stakeholder organizations supporting the US blood supply and transfusion‐dependent patient care, in liaison with the US Department of Health and Human Services, the Centers for Disease Control and Prevention and the Food and Drug Administration.
- For over 20 years, the IDTF has coordinated blood‐supply support for large‐scale natural disasters, epidemics and cyberattacks.

## INTRODUCTION

The founding of the Association for the Advancement of Blood & Biotherapies (AABB) (originally the American Association of Blood Banks) in 1948 was partly in response to a 1947 mass casualty event (MCE) [1]. A massive explosion of a fertilizer ship near Galveston, Texas, caused nearly 1800 hospitalizations. Lack of standardized blood collection and testing practices and communication channels hindered the blood support needed from other regions. At an ensuing meeting, blood bankers discussed problems revealed by this event and voted to establish the AABB as an association for communication, standard‐setting and representation for individuals, transfusion services and blood centres in the US blood banking community [1].

Unlike most other nations, there is no single organization in the United States that performs all blood collections. Blood components are collected by over 50 community blood centres and 90 hospital‐based blood centres [2]. Blood components are federally regulated by the Food and Drug Administration (FDA) in the US Department of Health and Human Services (HHS). However, prior to 2001 there was no overall framework for coordinating responses to emergencies affecting the US blood supply.

The AABB Interorganizational Task Force for Domestic Disasters and Acts of Terrorism (formal name shortened here to the Interorganizational Disaster Task Force [IDTF]) was founded in December 2001 in response to the 9/11 terrorist attack [3]. On 11 September 2001, four US airliners were hijacked. Two destroyed the twin towers of the World Trade Center in New York City, one was flown into the US Department of Defense headquarters, the Pentagon, in Washington, DC, and one was crashed in Pennsylvania. Nearly 3000 persons were killed. Because of the nature of the event, there were far more deaths than severe injuries. In New York City, 1100 patients came to emergency rooms, and 181 were admitted [4]. In the Washington area, 106 patients presented for care and 49 were admitted, including 10 to a burn centre [5, 6]. The total known red blood cell (RBC) transfusions were 436 units for (1) 230 admissions in New York City (224 units in first week) [7], (2) the Washington burn centre (178 units over lengthy hospitalizations) [6] and (3) other Washington hospitals (34 units) [8].

These modest transfusion needs were readily met by local blood suppliers. However, blood donation became one of the national responses to the tragedy, initially in the context of encouragement by national leaders, concern for additional attacks and temporary grounding of US aviation to prevent more hijackings, interrupting routine airborne blood shipments [9]. Over the next 2 months, up to 572,000 excess blood collections were given and an estimated 208,000 RBC units later expired [10]. Lack of a unified message on blood needs was decried [11].

On 11 December 2001, the AABB convened a new task force of blood banking and blood supply organizations and US government agencies to evaluate the blood community's response to 9/11 and develop recommendations for future disasters and acts of terrorism [3]. The IDTF's charges included improved coordination of blood collection and supply, improved coordination among blood organizations and governmental departments and development of a national blood‐supply response plan. Their recommendations addressed evaluation of blood needs, coordination of blood collections with needs, establishment of communication lines with federal agencies and identification of transportation sources for blood units and testing materials. Their recommendations were endorsed to the HHS in January 2002 by the HHS Advisory Committee on Blood Safety and Availability [3].

The purpose of this article is to review the objectives, organization and activities of the IDTF.

## MATERIALS AND METHODS

We reviewed the organizational structure and operational procedures of the IDTF and identified key activation events during the IDTF's tenure (F.B.). We performed a review of published and archival information about the formation and activities of the IDTF, examining Ovid MEDLINE (2001+), *AABB News* (2018+), the HHS national emergency response framework, and the initial IDTF report and recommendations (G.R.).

## RESULTS

### Task force objectives

The objectives of the IDTF are to (1) help ensure that blood collection efforts resulting from domestic disasters and acts of terrorism are managed properly, (2) deliver clear and consistent messages to the public regarding the status of America's blood supply and (3) identify infrastructure challenges and coordinate transport of blood. In the National Response Framework (Emergency Support Function #8, Public Health and Medical Services) of the Federal Emergency Management Agency within the US Department of Homeland Security (DHS), HHS provides liaison to the IDTF for logistical support and coordination of national public announcements about blood donation [12].

### Task force organization

AABB serves as the designated coordinating entity for the IDTF. The core members serve as the Critical Events Assessment Group (CEAG) (Figure 1). America's Blood Centers (ABCs) and the Blood Centers of America (BCAs) represent all US non–Red Cross blood centres. The American Red Cross (ARC) is the largest US blood provider and is also designated as a federal partner for humanitarian disaster relief. The Armed Services Blood Program (ASBP) collects blood for US military facilities and has vast experience providing transfusion needs for trauma patients. The ASBP also supports civilian blood needs in a domestic disaster. Each US civilian and military blood centre is a member of one of these four blood collection organizations: ABC, BCA, ARC or ASBP. HHS has a liaison role with the IDTF, monitors the national blood supply and disseminates public information. The CEAG serves as the initial evaluation group when a disaster occurs or may occur.

**FIGURE 1:**
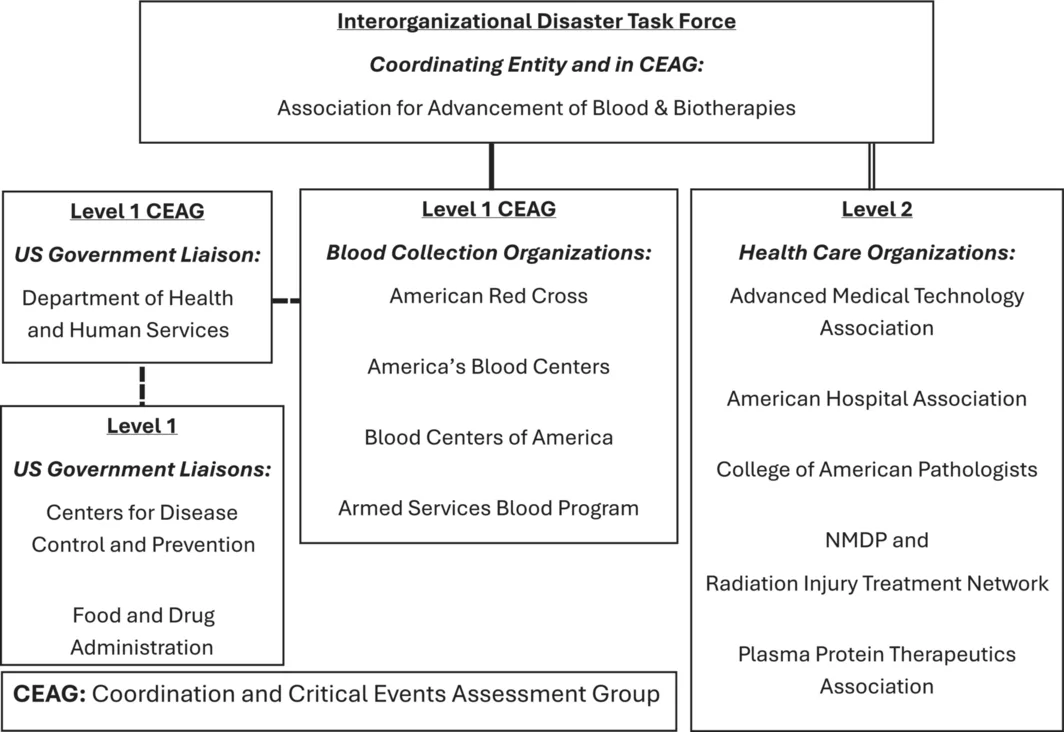
Association for the Advancement of Blood & Biotherapies (AABB) Interorganizational Disaster Task Force (IDTF) Organization. The IDTF is coordinated by the AABB. The Critical Events Assessment Group (CEAG)—the AABB and four civilian and military blood collection organizations (solid line) and the US Department of Health and Human Services (HHS)—evaluate emerging events. When needed, they consult the Centers for Disease Control and Prevention and the Food and Drug Administration in HHS. The government agencies serve in liaison with the IDTF (dotted lines). When the CEAG activates the entire IDTF for a disaster, five Level 2 health care organizations involved in transfusion support are notified and participate in monitoring and response (double line). NMDP, (Formerly) the National Marrow Donor Program.

Other IDTF members are shown in Figure 1. Two federal agencies within HHS are Level 1 liaison members along with the CEAG. The Centers for Disease Control and Prevention (CDC) addresses infectious disease issues in the blood supply. The FDA provides oversight for regulatory issues in emergency blood collection and testing. The CEAG consults these liaisons when needed, in initial evaluations.

When the IDTF is activated, five Level 2 member organizations involved with transfusion support and blood supplies are notified by AABB and brought into task force–wide communications to exchange information on disaster impacts and responses. The Advanced Medical Technology Association represents manufacturers of medical supplies, equipment and diagnostic tests needed by blood collection facilities. In the context of the IDTF, the American Hospital Association monitors impacts of disasters on healthcare delivery. The College of American Pathologists (CAP) represents many hospital transfusion service physicians and has a large laboratory accreditation programme. NMDP (formerly known as the National Marrow Donor Program) maintains the national registry for volunteer haematopoietic stem cell donors and co‐administers the Radiation Injury Treatment Network (RITN), which coordinates care for victims of radiologic events. The Plasma Protein Therapeutics Association represents manufacturers of plasma derivatives.

### Activity categories

#### Preparation

The IDTF performs and promotes several types of disaster preparations. Blood collection facilities are encouraged to prepare advance messaging templates to inform the media and the public whether blood donations are needed or not after an incident [13]. High‐profile events requiring extra security such as the United Nations General Assembly sessions and presidential inaugurations are designated as National Special Security Events (NSSEs) by the DHS [14]. On the eve of NSSEs, the IDTF evaluates regional and national blood supplies in case of emergency. An example of an IDTF NSSE planning report (2025 presidential inauguration) is available in Supporting Information: Item 1. DHS has conducted large‐scale National Level Exercises (NLEs) [15] biennially with mock scenarios such as earthquake, cyberattack and hurricane disasters, and the IDTF has participated in NLEs to support their activities and test task force processes. On its website, the IDTF recommends that blood centres raise their disaster priority status with state and local emergency management agencies. At the AABB annual meetings, IDTF representatives meet to review the past year, discuss current disaster‐related issues and review analyses of blood needs in MCEs [16, 17].

#### Communication

The IDTF prepares a weekly summary of the US blood supply for its members, including HHS; an example is available in Supporting Information: Item 2. The IDTF produced two editions of a disaster handbook for blood centres and transfusion services [13]. IDTF members wrote chapters on disaster management for the 16th and 17th editions of the AABB Technical Manual [18, 19]. One of us then on the IDTF (G.R.) organized a seminar at the 2017 AABB annual meeting with blood banking speakers from the 2013 Boston Marathon bombing and the 2015 Paris terrorist attack. In 2020 and 2021, IDTF leadership provided information on the impact of the COVID‐19 pandemic to the HHS Advisory Committee of Blood and Tissue Safety and Availability and submitted recommendations for improved assessment of the national blood supply [20, 21]. The monthly online publication *AABB News* frequently covers topics on disaster preparedness and IDTF activities (Table 1): most recently, in the feature focus of the February 2025 issue.

**TABLE 1:** *AABB News* articles related to disaster preparedness.

Topic	Date
Hurricane Harvey	January 2018
Mass casualties	August 2018
Disaster issue	January 2019
Military transfusion support	June 2019
COVID‐19 pandemic	July 2020
Cybersecurity	March 2021
Texas ship disaster 1947	January 2022
Disaster and trauma responses	May 2023
Hurricane Ian	October 2023
Military transfusion support	April 2024
Disaster issue	February 2025

#### Responses

For possible future disasters such as an advancing hurricane, the IDTF CEAG monitors the situation, assesses the regional and national blood supply and activates the task force when needed. When an unexpected disaster occurs, the IDTF handbook recommends that affected blood collectors assess the type of event and injuries, the expected hospital admissions and the potential impact on blood collections and inventories. If blood supply assistance is needed, the blood collector contacts the AABB IDTF office. The AABB rapidly convenes a conference call of the CEAG to determine whether IDTF activation is needed. If the CEAG blood organizations activate the IDTF, all Level 1 and Level 2 members are notified. Initial strategy and coordination include messaging to the blood community and the public and assessment of the need for blood transport to the affected region. Information channels are established with the affected region and IDTF conference calls are conducted 1–2 times a day to discuss blood supply support. IDTF representatives provide information to and from their organizations. The IDTF also liaises with HHS on the need for messaging to the public about blood donation needs; when indicated, both the IDTF and HHS issue statements encouraging blood donation. After the emergency is resolved and confirmation is received that all supply needs are being managed through routine channels, the IDTF issues an all‐clear to its member organizations and prepares and reviews an after‐action report to assess the response and improve future efforts.

### Task force responses—Overview

Disasters and emergencies are broadly characterized as natural from forces of nature or as human‐derived through intent, negligence or error [22]. Some examples of events involving the IDTF are given below.

#### Weather events

The most common events for activating the IDTF have been hurricanes and other large storms. Table 2 lists selected major US storms during the IDTF's tenure. Hurricanes typically impact the Gulf Coast and the US Southeast in the months of August to October. Storms disrupt donor collections via flooding, community structural damage, power outages, travel disruption and difficulties obtaining donor testing. The IDTF coordinates blood component shipments and fuel supplies for backup generators. Hurricanes Maria in 2017 and Helene in 2024 caused a major shortage of intravenous saline solutions by damaging critical pharmaceutical plants in Puerto Rico and North Carolina, respectively [23, 24]. In response to the shortage, hospitals reduced the use of saline solutions for flushing transfusion lines (AABB) and the FDA permitted emergency importation of intravenous solutions from other countries.

**TABLE 2:** Major US storms, 2002–2024.

Date	Storm	Region
August 2004	Charley	Florida, Southeast
July 2005	Dennis	Florida
August 2005	Katrina	Florida, Louisiana, Texas
September 2005	Rita	Florida, Louisiana, Texas
October 2005	Wilma	Florida
October 2012	Sandy	New York, East Coast
August 2017	Harvey	Texas
September 2017	Maria	Puerto Rico
October 2018	Michael	Florida, Southeast
August 2020	Laura	Louisiana, Texas
February 2021	Uri	National
August 2021	Ida	Louisiana
September 2022	Ian	Florida, Southeast
May 2024	Multiple	Central United States
September 2024	Helene	Florida, Southeast

*Note*: Weather events requiring IDTF activation mostly impacted the Gulf Coast and southeastern United States in the months of August through October.

Abbreviation: IDFT, Interorganizational Disaster Task Force.

#### Haiti earthquake, 2010

A severe earthquake in Haiti in 2010 destroyed the nation's only blood processing centre. The Haitian Red Cross requested blood supply assistance from the ARC [25]. The ASBP supplied blood to a US hospital ship serving as a trauma centre [26, 27]. The IDTF helped coordinate some of the blood supplies. This was the IDTF's only activation to date for an international event.

#### Zika virus epidemic—Puerto Rico, 2016

During a Zika virus epidemic in Puerto Rico in 2016, blood collections were halted for 1 month until donor nucleic acid testing was emergently approved and implemented [28]. During this period, the IDTF helped facilitate blood shipments from the US mainland.

#### COVID‐19 pandemic, 2020

Early in the COVID‐19 pandemic, the IDTF arranged for blood shipments to alleviate an acute blood shortage in Washington state in March 2020 [20]. As COVID‐19 cases declined and deferred surgeries and therapies resumed, periodic national blood shortages occurred. The IDTF sent out several messages calling on the public to donate blood when needed.

#### Cyberattacks, 2024–2025

Two large US blood centres suffered recent ransomware cyberattacks. OneBlood in the US Southeast and New York Blood Center Enterprises were each disrupted for 9–11 days in July 2024 and January–February 2025, respectively [29, 30]. Systems supporting blood collection and processing had to be taken offline. The IDTF coordinated emergency blood shipments from other blood suppliers in both incidents to maintain hospital patient care. In the OneBlood cyberattack, 20 different blood centres contributed approximately 3700 RBC units and 1400 plateletpheresis units. An outline of the OneBlood after‐event report showed how the IDTF helped mitigate a major interruption of regional blood supplies (Supporting Information: Item 3).

#### Mass casualty events

None of the largest MCEs in the United States since 2002 [16], such as the 2017 Las Vegas shooting (499 blood components transfused in 24 h [31]), have required IDTF blood support to date; local blood centres have met the needs without outside assistance. In the 2016 Orlando shooting (326 blood components in 24 h [32]), the regional blood centre OneBlood had issued an emergency donor appeal a few days previously because of a shortage. OneBlood contacted the IDTF shortly after the shooting, but local blood donations and assistance from nearby blood centres rapidly replenished supplies and the IDTF was not activated [33].

## DISCUSSION

The IDTF provides a useful forum for private and governmental partnership in response to major events threatening the national blood supply. The US blood supply system is decentralized across over 50 blood collection networks and facilities. However, on the IDTF, several organizations representing all community and military blood collection sites have worked together for the greater good to provide lifesaving blood transfusions in a crisis. Mutual coordination and support have overcome what could have been detrimentally fragmented communications and responses. Additionally, organizations on the IDTF representing hospitals, pathologists, vendors for blood bank equipment and plasma derivatives and a radiation treatment network provided status information and communication channels with their members.

Lessons learned include the need to pre‐plan with local and regional emergency agencies for recognition of blood collection facilities as critical to disaster responses. Consistent and unified communications and messaging during disaster responses are needed to keep the blood community and the public informed on the status of the blood supply and the need for blood donations. Blood centres have recognized that supply chain risk assessment must extend internationally for vital materiel such as blood filters for which the sources are limited [34]. The effects of the COVID‐19 pandemic were very complex, including blood shortages, staffing shortages and the need for convalescent plasma. COVID‐19 and recent cybersecurity events are reminders of the need for vigilance and adaptation to new and emerging threats to the blood supply [35].

The HHS liaison to the IDTF has historically been from the Office of Infectious Disease and HIV/AIDS Policy (OIDP) in the Office of the Assistant Secretary for Health of HHS (OASH) [36]. Under this arrangement, the Assistant Secretary for Health and HHS leadership had access to the IDTF's blood supply reports on a routine weekly basis and during disaster responses. The IDTF's other federal partners, including FDA, CDC and ASBP, were updated as needed. The current US administration has restructured OIDP and OASH as part of a wide‐ranging governmental reorganization. The IDTF remains in contact with its federal partners during this transitional period.

In conclusion, the AABB IDTF has gained over 20 years of US national experience in preparing for, communicating about and responding to disasters and would‐be disasters affecting blood supply and demand. The IDTF's cooperative partnership of blood organizations, key healthcare organizations and liaisons from federal agencies has successfully mitigated numerous threats to the blood supply from a range of events.

## CONFLICT OF INTEREST STATEMENT

The authors declare no conflicts of interest. G.R. is a former longtime IDTF member from the College of American Pathologists (CAP, 2003–2024), and F.B. is the AABB's IDTF director (2012–present).

## Supporting information


**Data S1.** Supporting information.

## Data Availability

Data sharing is not applicable to this article as no new data were created or analysed in this study.
