# Supplementary material for: An interorganizational task force for disasters affecting US blood supply
Source: Vox Sang. 2025 Dec 9;121(10):1565–71. doi: 10.1111/vox.70166 (PMC13649364; doi:10.1111/vox.70166)
Supplement: Supplementary file 1 [file VOX-121-1565-s001.docx]

**Supplemental Material: 3 Items**

**Item 1**

**National Special Security Event (NSSE) Planning Report - Sample**

2025 Presidential Inauguration

1) Description of Event
The inauguration of Donald Trump as the 47th president of the United States will take place on Monday, January 20, 2025, on the West Front of the United States Capitol in Washington, DC. The event will include a swearing-in ceremony, a signing ceremony, an inaugural luncheon, a pass-in-review, a procession, and a parade. Inaugural balls are held at various venues before and after the inaugural ceremonies. This event has been designated as a National Security Special Event (NSSE) by the Department of Homeland Security for the period of January 15 – 22, 2025.

2) Threat Assessment
At the present time there are no known credible threats to the event.

3) Major Blood Suppliers

Primary Suppliers DC Region:

American Red Cross (ARC) Distribution Centers – Baltimore MD and Richmond VA; Inova Blood Donor Services - Sterling, VA

Backup Suppliers DC Region:

New York Blood Center; ARC Philadelphia PA & Johnstown PA Centers; Rhode Island Blood Center; Vitalant New England

4) Major Trauma Hospitals
Primary DC Region: George Washington University Hospital, MedStar Washington Hospital Center

Secondary DC Region: Howard University Hospital, Sibley Memorial Hospital, Inova Fairfax Hospital

-Government Facilities: Walter Reed National Military Medical Center

5) Current and Projected Blood Supply Current National Blood Supply as of 01/16/25: separate file

Current Local and Supporting Facility Blood Supply as of 01/16/25
Red Cells: Sufficient supply

Platelets: Sufficient supply

Plasma: Sufficient supply

6) Response Strategies

ARC Distribution Centers

Primary – Baltimore, MD, backup locations – Philadelphia, PA, Richmond, VA. ARC has adequate blood supply in all three locations to assist in any emergency or incident that may occur between January 15th and January 22nd. These sites are adequately staffed to respond to any emergency blood needs.

Armed Services Blood Program

The Blood Center at Walter Reed National Military Medical Center (Bethesda, MD) has sufficient blood supplies to support its day-to-day operations (inventory level is green as of 15 January 25). The ASBP will stand by and provide assistance if needed.

Inova Blood Donor Services

New York Blood Center (NYBCe)
Vitalant

NYBCe, Vitalant, and Inova have sufficient staffing and inventory in place across all operating areas to support any emergency efforts that may arise.

**Item 2**

**Weekly U.S. Blood Supply Status Reports - Samples for Yellow or Red Status**

[Green, yellow or red highlighting is used to help denote supply status]

*********************************************************************************

**AABB Interorganizational Disaster Task Force - Weekly Update on Status of the U.S. Blood Supply – August 21, 2024: Yellow (1-2 day supply)**

*Blood supply status definitions:*

*Green (3+ day supply): The majority of blood center inventories have enough blood to meet normal demands.*

***Yellow (1-2 day supply): The majority of blood center inventories are running low and need blood donations.***

*Red (less than 1 day supply): The majority of blood center inventories are critically low on blood and need donations as soon as possible.*

*This is for Type O Red Blood Cell inventories

**Blood has a short shelf life and must be continually replenished. Therefore, regardless of the status of the U.S. blood supply, it is important to encourage healthy, eligible individuals to donate blood.

**Statement**
Our nation’s blood supply is lower than it should be. All individuals who are healthy and able to donate should contact their local blood center to make an appointment. Donating blood is a safe process and donors are needed to help save lives.

*********************************************************************************

**AABB Interorganizational Disaster Task Force - Weekly Update on Status of the U.S. Blood Supply  -  February 16, 2022: RED (less than 1 day supply)**

*Blood supply status definitions:
Green (3+ day supply): The majority of blood center inventories have enough blood to meet normal demands.*
*Yellow (1-2 day supply): The majority of blood center inventories are running low and need blood donations.*
***Red (less than 1 day supply): The majority of blood center inventories are critically low on blood and need donations as soon as possible.***

*This is for Type O Red Blood Cell inventories
**Blood has a short shelf life and must be continually replenished. Therefore, regardless of the status of the U.S. blood supply, it is important to encourage healthy, eligible individuals to donate blood.

**Statement**
Our nation’s blood supply is dangerously low. We urge all individuals who are healthy and able to donate to contact their local blood center immediately to make an appointment. Your donation is critical to ensuring blood is available for all those in need.

**Item 3**

**After-Action Report - Sample**

**AABB Interorganizational Disaster Task Force (IDTF) Response to Blood Center Ransomware Cyberattack, July-August 2024**

Ransomware Attack Onset

July 29, 2024: OneBlood discovered ransomware attack

Initial Alert and IDTF Assessment

July 30-31, 2024: OneBlood informed IDTF and announced ransomware attack

Critical OneBlood information systems were taken offline in defense

Switch to manual procedures

Hospitals advised to prepare for potential blood shortages

IDTF Critical Events Assessment Group (CEAG) convened immediate conference call and activated IDTF

Activation and Coordination

CEAG began daily morning conference calls and set up dedicated e-mail thread

Entire Task Force notified

IDTF public relations subgroup prepared public donation messaging for dissemination by AABB

Operational Support and Recovery

IDTF coordinated shipment of immediate critical blood component needs

Platelets and group O D+ and D-negative RBCs

IDTF monitored OneBlood operational capacity

Labeling and blood distribution challenges

IDTF updated key stakeholders including Florida Hospital Association

Strategic Planning

IDTF obtained forecasted blood demands, including post-attack ‘catch-up’ surge

Concurrent approaching Hurricane Debby was also monitored for need for additional support

Conclusion of Support

OneBlood systems were restored

August 7, 2024: no further blood supply support needed

Final Monitoring and Deactivation

IDTF monitored OneBlood status and Hurricane Debby

August 6-10: Florida category 1 hurricane, then East Coast subtropical storm

August 10, 2024: full deactivation of IDTF

After-Action Report

IDTF prepared and reviewed after-action summary and evaluation

All patient blood needs were ultimately met

Prevention of disruption to national blood supply
